# Editorial: Biotechnology of Microalgae, Based on Molecular Biology and Biochemistry of Eukaryotic Algae and Cyanobacteria

**DOI:** 10.3389/fmicb.2017.00118

**Published:** 2017-02-01

**Authors:** Takashi Osanai, Youn-Il Park, Yuki Nakamura

**Affiliations:** ^1^School of Agriculture, Meiji UniversityKawasaki, Japan; ^2^Department of Biological Sciences, Chungnam National UniversityDaejeon, South Korea; ^3^Institute of Plant and Microbial Biology, Academia SinicaTaipei, Taiwan

**Keywords:** *Chlamydomonas*, *Chlorella*, cyanobacteria, metabolic engineering, microalgae, photoreceptor, *Synechocystis*, triacylglycerol

Microalgae, including eukaryotic algae and cyanobacteria, comprise a diverse group of microscopic unicellular photosynthetic organisms that inhabit almost all ecological niches. Recently, energy and resource production using microalgae have received a great deal of attention (Banerjee et al., 2016). Several species of microalgae are commercially available. They include *Arthrospira platensis, Chlorella* species, and *Euglena gracilis* (Koller et al., 2014; Gouveia et al., 2016; Yamada et al., 2016). Microalgae produce high-value products such as pigments, lipids, bioplastics, carbohydrates, and amino acids (Minhas et al., 2016). However, production by microalgae is generally less efficient than current industrial production. To increase productivity, a basic understanding of photosynthesis, metabolism, and the cellular structure of microalgae using molecular genetics is indispensable. Here, we introduce current researches, which cover the basic and applied science of eukaryotic algae and cyanobacteria.

The study of the primary carbon metabolism of photosynthetic organisms has a long history, and basic scientists have devoted their time to deciphering the regulatory mechanisms of primary metabolism and the biochemical properties of metabolic enzymes. Basic scientists have studied the regulator mechanisms of anabolic carbon processes such as carbon fixation, gluconeogenesis, and glycogen/starch production. However, the regulatory mechanisms of the catabolic processes of microalgae have been less well studied. The downstream metabolites of sugar catabolism are the precursors of biofuels and bulk chemicals, and an understanding of metabolic sugar regulation would therefore lead to improvements in productive capacity and chemical variety (Oliver et al., 2016). Unicellular cyanobacteria, such as *Synechocystis* and *Synechococcus* species, and filamentous cyanobacteria, such as *Nostoc* species, are the preferred microbial cell factories because they are easy to genetically manipulate (Atsumi et al., 2009; Khanna and Lindblad, 2015). Recently, there has been an increase in the number of publications dealing with the metabolic engineering of cyanobacteria. Several transcriptional regulators that activate the gene expression of sugar catabolic enzymes in cyanobacteria have been identified in *Synechocystis*; for example, an RNA polymerase sigma factor (SigE) and a response regulator (Rre37) (Osanai et al., 2011, 2014). Overexpression of these genes causes increased production of polyhydroxybutyrates, which are bioplastic polyesters (Osanai et al., 2014). Therefore, the elucidation of the regulatory mechanism of primary carbon metabolism would be important.

An understanding of photosynthetic electron transport systems and light-to-chemical energy conversion is indispensable for biorefineries. Varying light conditions mean that photoautotrophs must sense light intensity, quality, and light/dark photoperiods to carry out optimal photosynthesis (Eberhard et al., 2008). Near-UV light, which causes damage to the photosynthetic apparatus, is effectively screened-out by light-blocking pigments such as anthocyanin and other near-UV absorbing pigments. The application of multiple interdisciplinary approaches has expanded our knowledge of the structure and function of traditionally well-characterized photoreceptors and their downstream signaling mechanisms. The last 10 years in particular have witnessed discoveries in the newly emerging field of plant photobiology, especially bilin photoreceptor phytochromes (Rockwell and Lagarias, 2010).

Various types of cyanobacterial phytochromes have been discovered using genomic information from *Synechocystis*, and heterocystous (*Nostoc punctiforme*) and non-heterocystous (*Microcoleus* IPPAS B353) cyanobacteria; they are distinguished by their domain structures and by the number of cysteine (Cys) amino acid residues they contain, which play a role in the attachment of tetrapyrrole chromophores. Cyanobacteria are able to sense near-UV and the entire visible light range using various bilin photoreceptors. Their biological functions are implicated in positive and negative phototaxis, state transition, and salt acclimation, and to a lesser extent in other photobiological responses such as carotenoid accumulation and filament stacking (Wiltbank and Kehoe, 2016). Moreover, photosynthesis is regulated by photoreceptors such as phototropin from the green algae *Chlamydomonas reinhardtii* (Petroutsos et al., 2016) and cyanobacterial phytochromes Cph2 from *Synechocystis* (Schwarzkopf et al., 2014); the discovery of these photoreceptors has paved the way for the study of the coevolution of light-sensing and signaling pathways. Some eukaryotic algae contain phytochromes that are responsive to the visible spectrum, rather than to canonical red/far red light (Rockwell et al., 2014), which is a fascinating aspect of phytochrome evolution. Basically, cyanobacterial bilin photoreceptors transduce signals to the immediate downstream component via phosphorylation. These phosphorylated components often act as transcriptional factors, activating the downstream cascade related to transition state and phototaxis. Using these two-component systems, optogenetic approaches can be applied to selectively regulate gene expression for nutrient uptake and augment particular metabolic pathways in the field of biotechnology (Narikawa et al., 2015).

The study of the regulatory mechanisms that maintain cellular structure is also important for both basic and applied science. Lipids are the major constituents of cellular membranes (Li-Beisson et al., 2016). More specifically, microalgae contain thylakoid membranes, which are the sites of photosynthetic reactions that require the absorption of light energy to split water, and produce oxygen, ATP, and reductants. Moreover, lipids can be converted into biofuels. Microalgae are known for their high capacity for lipid production. Triacylglycerols and other neutral lipids are the major form of storage lipids; they are often termed “oils,” and represent an inert end-product of photosynthetic carbon assimilation. Compared with the higher plants in which storage lipid accumulation only occurs in limited types of tissues/cells, unicellular microalgae have a high degree of flexibility that alters cellular lipid metabolic flux (i.e., lipid biosynthesis for photosynthetic membrane constructions such as in plant leaves, or lipid accumulation for storage in plant seeds) during growth. For instance, nitrogen starvation alters the growth phase of algae and induces oil accumulation at the expense of cellular membrane lipids in many algal species. Thus, flexible lipid metabolic flux in unicellular systems, overall high lipid productivity, photosynthetic capacity that allows carbon fixation, and rapidly growing gene manipulation technologies, have promoted the use of microalgae as an emerging synthetic platform in metabolic engineering. A basic understanding of complex algal lipid metabolism will guide us to the most feasible and effective strategies for creating biofuels.

Therefore, microalgae have great potential for biofuel and biomaterial production. However, this translational research requires knowledge of molecular biology and biochemistry. Therefore, we believe that these researches contribute to crossing the “valley of death” (the gap between basic research and commercialization) in the future.

## Author contributions

All the authors of this manuscript confirmed their contribution and approved it for publication.

### Conflict of interest statement

The authors declare that the research was conducted in the absence of any commercial or financial relationships that could be construed as a potential conflict of interest.
